# Effects of personalized live-remote exercise for individuals living beyond primary curative cancer treatment: study protocol for a multinational, super umbrella randomized controlled trial (LION-RCT)

**DOI:** 10.1186/s13063-025-09263-1

**Published:** 2025-11-24

**Authors:** Anouk E. Hiensch, Jana Müller, Eva M. Zopf, Martina E. Schmidt, Evelyn M. Monninkhof, Mark Trevaskis, Jon Belloso, Dorothea Clauss, Nadira Gunasekara, Ana Joaquim, Sofia Viamonte, Moritz Schumann, Lars Heinrich, Alina Kias, David Binyam, Lonneke van de Poll, Laurien M. Buffart, Neil K. Aaronson, Elsken van der Wall, Anna Campbell, Joachim Wiskemann, Helene Rundqvist, Alberto J. Alves, Ander Urruticoechea, Wilhelm Bloch, Martijn M. Stuiver, Yvonne Wengström, Karen Steindorf, Anne M. May

**Affiliations:** 1 Julius Center for Health Sciences and Primary Care, University Medical Center Utrecht, Utrecht University, PO Box 85500, Utrecht, GA 3508 The Netherlands; 2 Department of Medical Oncology, Exercise Oncology Research Group, Heidelberg University Hospital, Medical Faculty Heidelberg, Heidelberg University, National Center for Tumor Diseases Heidelberg, a partnership between DKFZ and Heidelberg University Hospital, Heidelberg, Germany; 3 Cabrini Cancer Institute, Cabrini Health, Melbourne, VIC Australia; 4 Mary MacKillop Institute for Health Research, Australian Catholic University, Melbourne, VIC Australia; 5 Division of Physical Activity, Cancer Prevention and Survivorship, German Cancer Research Center (DKFZ) and National Center for Tumor Diseases (NCT) Heidelberg, a partnership between DKFZ and University Medical Center Heidelberg, Heidelberg, Germany; 6 Gipuzkoa Cancer Unit, OSID-Onkologikoa, BioGipuzkoa, Osakidetza, San Sebastian, Spain; 7 Institute for Cardiovascular Research and Sports Medicine, German Sport University Cologne, Cologne, Germany; 8 ONCOMOVE®-Associação de Investigação de Cuidados de Suporte em Oncologia (AICSO), Vila Nova Gaia, Portugal; 9 Unidade Local de Saude Gaia Espinho, Vila Nova de Gaia, Portugal; 10 Department of Sports Medicine and Exercise Therapy, Chemnitz University of Technology, Chemnitz, Germany; 11 Medical Faculty, University of Heidelberg, Heidelberg, Germany; 12 Department of Psychosocial Research and Epidemiology, Netherlands Cancer Institute, Amsterdam, The Netherlands; 13 Department of Medical BioSciences, Radboud University Medical Center, Nijmegen, The Netherlands; 14 School of Applied Science, Edinburgh Napier University, Edinburgh, Scotland UK; 15 Karolinska Institutet and Karolinska Comprehensive Cancer Center, Karolinska University Hospital, Stockholm, Sweden; 16 University of Maia, Research Center in Sports Sciences, Health Sciences and Human Development, Maia, Portugal

**Keywords:** Exercise, Cancer, Online live-remote, Fatigue, Quality of life, Emotional distress, Physical functioning, Peripheral neuropathy, Randomized controlled trial

## Abstract

**Background:**

Exercise is an effective strategy to reduce cancer- and treatment-related side effects and improve quality of life (QoL). Larger exercise effects are observed in cancer survivors with a higher symptom burden and when exercise interventions are supervised. Most studies conducted to date have not screened for symptoms at baseline and tailored the exercise intervention accordingly. Additionally, time and travel distance are common barriers to participation in supervised in-person exercise programs. Live-remote exercise, where exercise sessions are supervised by an exercise professional via a videoconferencing platform, might help overcome these barriers. Here, we describe the design of the LION randomized controlled trial (RCT). This RCT aims to assess the (cost-)effectiveness of side effect-targeted, live-remote exercise on QoL and the participants’ most burdensome side effect—fatigue, emotional distress, low physical functioning, or chemotherapy-induced peripheral neuropathy (CIPN)—in individuals who have completed primary curative cancer treatment.

**Methods:**

The LION study is a multinational RCT that will enroll 352 individuals who have completed primary curative cancer treatment including chemotherapy, within the previous 12–52 weeks and screen positive for ≥ 1 of the targeted side effects. Participants are randomly allocated (1:1) to the intervention or wait list control group. Participants in the intervention group receive a 12-week supervised exercise program consisting of three live-remote exercise sessions per week. Each participant receives the same base module (2×/week) and one specific module (1×/week) targeting their most burdensome side effect. Wait list control participants receive the same exercise program 12 weeks post-baseline. The primary outcomes are HRQoL (EORTC QLQ-C30 summary score) and a standardized symptom score based on each participant’s most burdensome side effect (physical fatigue: EORTC QLQ-FA12, emotional distress: PHQ-ADS, physical functioning: EORTC QLQ-C30 modified physical functioning scale, CIPN: EORTC QLQ-CIPN20), assessed at baseline, 6, 12 (primary time point), 18 (wait list control group only), 24 and 36 weeks post-baseline.

**Discussion:**

This RCT will provide evidence regarding the (cost-)effectiveness of side effect-targeted, live-remote exercise in individuals experiencing side effects following primary curative cancer treatment. If proven (cost-)effective, live-remote exercise could be offered to individuals as part of standard follow-up cancer care to extend the reach of exercise support.

**Trial registration:**

NCT06270628. Registered on February 13, 2024.

**Supplementary Information:**

The online version contains supplementary material available at 10.1186/s13063-025-09263-1.

## Introduction

Many people living beyond primary curative cancer treatment suffer from cancer- and treatment-related side effects like fatigue, emotional distress, low physical fitness, and chemotherapy-induced peripheral neuropathy (CIPN) [1–3]. These side effects strongly affect health-related quality of life (HRQoL) of an increasing number of cancer survivors [4]. Hence, there is a compelling need for innovative supportive care strategies that improve their HRQoL.

Many studies have provided convincing evidence for the beneficial effects of exercise during and after curative cancer treatment on side effects and HRQoL [5, 6], resulting in the development of international exercise guidelines for cancer survivors [7, 8]. While these guidelines recommend exercise as a successful strategy to increase HRQoL and reduce symptom burden, several knowledge gaps have been identified.

First, to date, most research has focused on breast and prostate cancer survivors, which limits the generalizability of the findings to other cancer types and/or stages. Second, studies generally have not screened for the presence of (long-term) side effects at baseline and tailored the exercise intervention accordingly. Supervised exercise has proven to be more effective than unsupervised exercise for most side effects [9–11]. However, two of the most common barriers to attending and complying with supervised exercise are travel distance and time [12, 13]. In addition, the availability of training facilities that provide high-quality exercise interventions is limited, restricting their accessibility and reach. One promising approach to resolving these barriers is to deliver the intervention live-remote, where exercise professionals supervise entire exercise sessions via a videoconferencing platform in real-time, with live instructions to an individual or a group. The objective is to ensure high-quality exercise training in a home-based setting by providing online supervision.

Based on the results of three reviews, which included (non-)randomized studies with small sample sizes and cohort studies, live-remote exercise appears to be safe and feasible [14–16]. Some studies even suggest better attendance and retention rates compared to in-person supervised exercise. Although improvements have been observed in physical fitness and functional outcomes, effects on patient-reported outcomes have been inconsistent. High-quality and adequately powered studies are needed to establish the (cost-)effectiveness and safety of live-remote exercise programs for cancer survivors.

The multinational randomized controlled LION study is designed to demonstrate the (cost-) effectiveness of a side effect-targeted, live-remote exercise program on the following: (1) HRQoL and (2) the participant’s most burdensome side effect, including fatigue, emotional distress, perceived low physical functioning in daily life, or CIPN. A super umbrella design is used, which allows for the evaluation of several exercise regimens (based on participant’s most burdensome side effect) in a wide variety of individuals who have completed primary curative cancer treatment. Here, we describe the LION-RCT study protocol.

## Methods

### Study design

The LION study is a multinational randomized controlled trial (RCT) with two study groups: (1) an intervention group that receives a 12-week personalized, live-remote exercise program, and (2) a wait list control group that receives the same exercise program after the 12-week “control” period. Both groups will continue to receive usual care throughout the study period. The primary hypothesis is that participation in the 12-week live-remote exercise program will improve HRQoL and/or reduce participants’ most burdensome side effects at 12 weeks compared to the wait list control group.

This RCT is conducted at eight hospitals and study centers in five European countries and Australia: the Netherlands (University Medical Center Utrecht (UMCU, Sponsor) and the Netherlands Cancer Institute (NKI)), Germany (Heidelberg University Hospital/German Cancer Research Center (DKFZ)/National Center for Tumor Diseases (NCT) Heidelberg and German Sport University Cologne (DSHS)), Portugal (Associação de Investigação de Cuidados de Suporte em Oncologia (AICSO) and Unidade Local de Saude Gaia Espinho), Spain (Gipuzkoa Cancer Unit, OSID-Onkologikoa, BioGipuzkoa, Osakidetza (ONK)), Sweden (Karolinska Institutet (KI)), and Australia (Cabrini Health and Australian Catholic University (ACU)).

The study protocol was approved in December 2023 by the institutional review board of the University Medical Center Utrecht, the Netherlands, and subsequently by the local ethical review boards of all participating institutions. Written informed consent will be obtained from all participants. The study was registered with ClinicalTrials.gov on 13 Feb 2024 (NCT06270628). The first participant was included on 14 Feb 2024.

More information about organizational aspects of the trial can be found in Additional file 1.

### Study sample

We plan to include 352 individuals who are approached within 12 to 52 weeks after completing primary cancer treatment, independent of their primary cancer diagnosis. To be eligible to participate in the study, individuals must meet the criteria outlined in Table 1. Medical in- and exclusion criteria are checked by an involved physician at the treating hospital.

**Table 1:** In- and exclusion criteria of the LION-RCT

**Inclusion criteria**	**Exclusion criteria**
• Age ≥ 18 years • Diagnosed with any type of invasive or hematological cancer • Completed primary cancer treatment with curative intent • Received systemic chemotherapy (or a stem cell transplantation in case of a hematological malignancy) as part of primary cancer treatment^a^ • For individuals undergoing endocrine, targeted, or immunotherapy, their maintenance treatment is not scheduled to be discontinued within the next 6 months • No evidence of distant metastatic disease (in case of solid tumors) • ECOG performance status ≤ 2 • Presence of at least one of the following side effects^b^: - Fatigue - Emotional distress - Perceived low physical functioning in daily life - Chemotherapy-induced peripheral neuropathy (CIPN) after having received neurotoxic chemotherapy • Access to stable internet connection • Able and willing to perform the exercise program and wear an activity tracker • Able to read, speak and understand the main country language	• Too physically active (i.e., > 150 min/week of moderate-to-vigorous intensity exercise) or participation in an exercise program comparable to the LION exercise program • Participated in a structured exercise intervention comparable to the LION exercise program during cancer treatment • Received chemotherapy for a previous cancer diagnosis within the past 5 years, or is experiencing ongoing side effects from previous chemotherapy regardless of when it was administered^c^ • Unable to complete the assessments or exercise sessions • Unstable on psychotropic medication^d^ • Any contraindications for exercise, including: - Severe neurologic or cardiac impairment according to ACSM criteria [17] - Uncontrolled severe respiratory insufficiency or dependence on oxygen suppletion in rest or during exercise - Uncontrolled pain • Any circumstances that would impede the ability to provide informed consent or adhere to the study requirements • Unable to attend the exercise program for more than 1 week

^a^This also includes new treatment regimens, including antibody drug conjugates

^b^The presence of these side effects is assessed using questionnaires: EORTC QLQ-C30 fatigue symptom scale, PHQ-ADS, EORTC QLQ-C30 physical function scale, 2 items from the PRO-CTCAE, respectively

^c^This exclusion criterion was updated in October 2025. Original exclusion criterion: received chemotherapy for a previous diagnosis

^d^This exclusion criterion was updated in October 2025. Original exclusion criterion: following or planning to follow a structured psychological intervention (e.g., cognitive behavioral therapy) during the intervention period, or unstable on psychotropic medication

### Recruitment and randomization

**Fig. 1:**
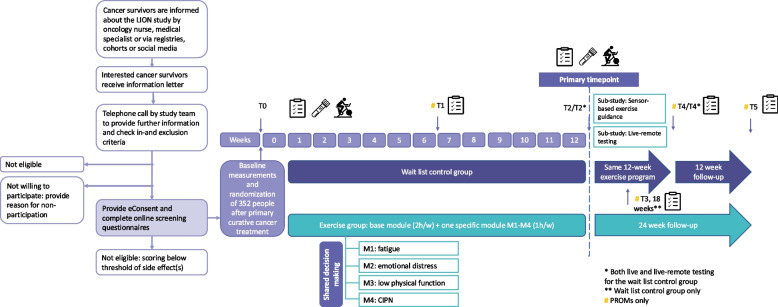
Recruitment and LION-RCT procedures

Participants are recruited in the abovementioned hospitals and study centers. Some centers have engaged additional recruitment sites. The study procedures are summarized in Fig. 1. Potentially eligible individuals are informed about the study by a member of the medical team (e.g., oncology nurse or medical specialist) during a regular follow-up visit or by email/letter/telephone/online call. In addition, national cancer registries/cohorts and social media (e.g., of national/local patient organizations) are used for recruitment. Interested individuals receive an information letter explaining the study aims and procedures. Subsequently, they are contacted by telephone to provide further information, answer questions and to check the inclusion and exclusion criteria. Potentially eligible individuals who choose not to participate in the LION-RCT are asked, but not required, to provide a reason for non-participation. Potentially eligible individuals who are willing to participate are asked to obtain exercise clearance for participation in the study from their treating physician or general practitioner. Once clearance is given, they are asked to provide online informed consent via Castor eConsent (Castor, The Netherlands). After providing consent, they complete online screening questionnaires to assess the presence and severity of the four side effects being targeted in this study.

To be eligible for the study, potential participants have to score above the threshold (Table 2) for at least one of the targeted side effects. Individuals who do not score above the threshold for any of the side effects are provided with country-specific resources related to exercising following a cancer diagnosis. Individuals who score above the threshold for multiple side effects meet with a member from the local research team to identify their most burdensome side effect through a shared decision-making process. Following this process, eligible participants are invited for their baseline visit (i.e., assessment of anthropometrics and physical fitness/performance, and blood draw).

**Table 2:** Pre-defined cut-off values for the side effects

**Side effect**	Questionnaire	Cut-off value
**Fatigue** [18]	EORTC QLQ-C30 fatigue symptom scale	> 39
**Emotional distress** [19]	PHQ-ADS	≥ 20
**Physical function** [18]	EORTC QLQ-C30 physical function scale	< 83
**CIPN**^**a**^** [**20]	PRO-CTCAE^b^	> 0

^a^Participants need to have been exposed to neurotoxic chemotherapy

^**b**^This questionnaire is used for eligibility screening only

After the baseline visit, participants are randomly allocated (1:1) to either the intervention or wait list control group by central computerized randomization using a blocked computer-generated sequence, effectively concealing the random allocation sequence. Castor EDC®, a cloud-based clinical data management platform, is used for randomization and data capture. Randomization is stratified by the participant’s most burdensome side effect, country and sex. Due to the nature of the intervention, blinding of participants and the study team to intervention assignment is not possible. However, data analysts will remain blinded to group allocation until the primary analyses have been completed. Participants randomized to the intervention group receive exercise instructions and an exercise kit at the end of their baseline visit. This kit includes the necessary exercise equipment to set-up their home exercise space: gym mat, dumbbell set with adjustable weights, resistance bands and an aerobic stepper. The wait list control group receives this kit 12 weeks post-baseline. All participants receive an activity tracker (Fitbit) at baseline.

### Live-remote exercise program

The 12-week live-remote multimodal exercise program is delivered via Zoom. Each participant receives three exercise sessions per week. The sessions are broadcasted from one center in each participating country and supervised by a qualified exercise professional. To ensure standardization of the exercise program across broadcast centers, a train-the-trainer workshop was delivered. These workshops have been recorded and are part of the general study onboarding to ensure that staff recruited during the course of the study receives the same training.

**Fig. 2:**
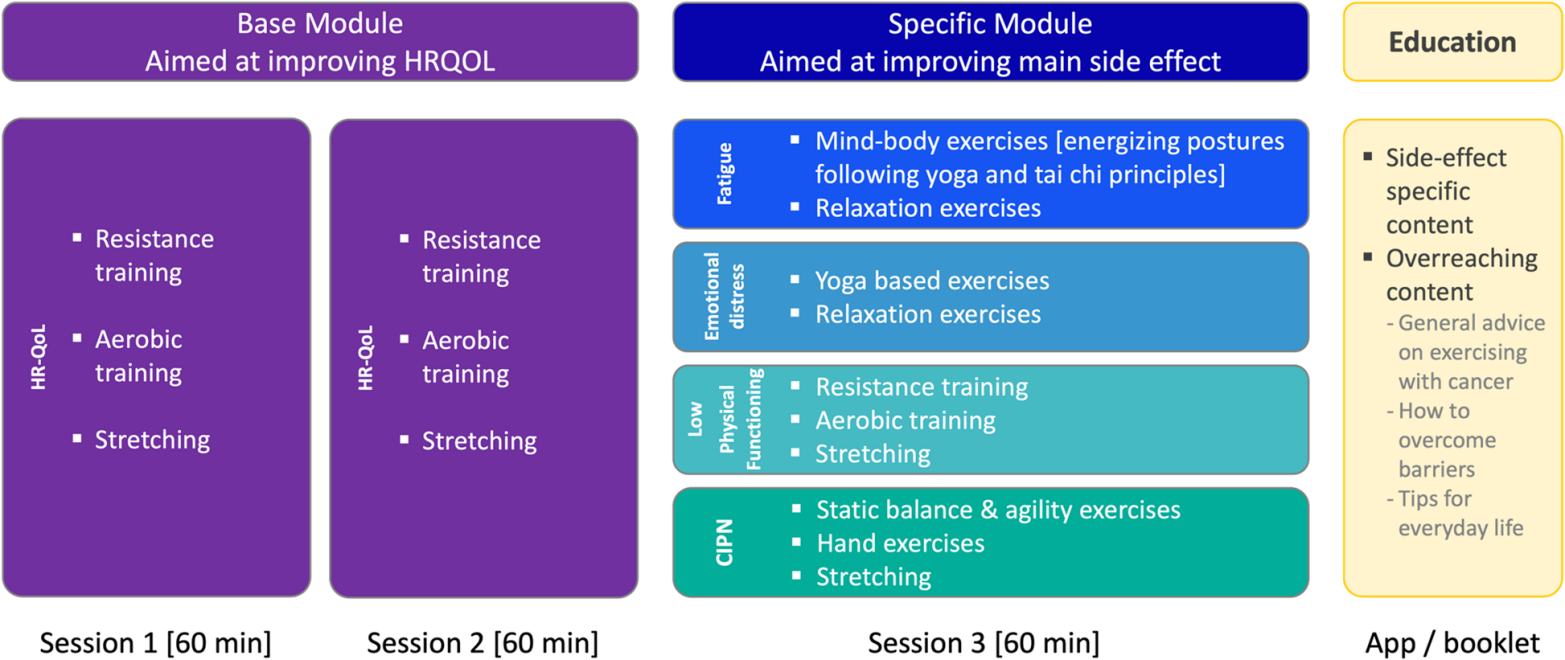
Overview of the base and specific modules of the LION exercise program

Several sessions are offered at various fixed times per week, lasting 60–75 min each and involving a maximum of 10 participants. Exercise sessions open 15 min before the start of the session to facilitate social interaction. To tailor the intervention to the individual participants, a modularized approach is used (Fig. 2). Each participant attends three exercise sessions per week: two sessions focused on improving HRQoL (i.e., Base Module) and one session targeting the participant’s most burdensome side effect (i.e., Specific Module), as detailed further below. Participants are advised to be as active as possible on all remaining days of the week. At the start of the exercise program, participants receive the multi-language PREFERABLE app. This app is linked to the activity tracker and was initially developed to support unsupervised exercise in patients with cancer in the previous PREFERABLE-EFFECT study (NCT04120298) [21]. Originally, the app only included exercises that participants learned during the exercise program and can be performed at home. Some adaptations have been made to the app to suit the needs of the LION-RCT (i.e., adding educational resources and audio files for relaxation exercises in the Specific Modules). This app provides exercise support beyond the exercise program, during holidays and after the end of the intervention.

Prior to the first session of the live-remote exercise program, an exercise professional conducts a one-on-one live-remote intake session with each participant, during which the exercises, training equipment, and training documentation are explained. In addition, the exercise environment, technical connection, and relevant medical history of the participant are checked. As part of the safety plan, the participant is educated about exercising safely at home. The training location and emergency contact details are documented, and a flowchart outlining the process for requesting assistance in case of an emergency is also discussed. Participants are encouraged to invite family members, informal caregivers, and/or friends to the intake session, allowing them to ask questions and provide support as needed. To ensure continuity and safety during the live-remote exercise sessions, back-office assistance (e.g., administrative staff, research staff or clinical staff not involved in delivering the session) is available to all exercise professionals. This person can assist the exercise professional by joining the session or contacting a patient offline if, for example, an adverse event or technical issue occurs. Additionally, break-out rooms are available via Zoom, which allows the exercise professional or back-office assistant to discuss medical information or other sensitive issues with the participant in private.

#### Base module—HRQoL

The base module is based on the recent ACSM exercise guidelines for cancer survivors [7]. Each session starts with a 5–10 min warm up, consisting of mobilization and light intensity aerobic exercises. The warm-up is followed by 30 min of resistance training, during which seven to eight exercises that target the major muscle groups are performed using either one’s own body weight, dumbbells or resistance bands. Two sets of 8–12 repetitions per exercise are performed at moderate-to-vigorous intensity (rate of perceived exertion (RPE) 13–17). Each time the maximum number of repetitions is reached in both sets, and the participant’s condition allows, the intensity of the exercise is increased. Conversely, the intensity is reduced if the participant does not reach the minimum number of repetitions or in case of health problems. After the resistance training component, participants perform 20 min of continuous aerobic training, comprised of step aerobics at a moderate intensity (65–75% of age-predicted maximal heart rate; RPE 12–13). The intensity is monitored with the activity tracker and the RPE scale. Finally, a 5–10 min cool down consisting of stretching exercises is performed to conclude each session.

#### Specific module—fatigue

Participants who report fatigue as their most burdensome side effect receive this module as their third exercise session per week. Each session starts with breathing and joint mobilization exercises (10 min). The core of the session (40 min) consists of mind–body exercises with a focus on body-awareness. The mind–body exercises include postures and movements based on yoga and Tai Chi, as these exercise modalities have been proven to have a beneficial effect on cancer-related fatigue [22]. To make it easier for the participant to follow and fully immerse themselves in the movement, the same sequence of mind–body exercises is performed every session. Each session concludes with a relaxation exercise (body scan) for 10 min.

#### Specific module—emotional distress

Participants who report emotional distress as their most burdensome side effect receive this module as their third exercise session per week. Each session starts with breathing and joint mobilization exercises (10 min). The core of the session (35 min) consists of yoga-based exercises with a focus on body-awareness. Yoga-based exercises are included as prior evidence supports yoga as a beneficial exercise modality to reduce anxiety and depressive symptoms in cancer survivors [23]. To make it easier for the participant to follow and fully immerse themselves in the movement, the same sequence of exercises is performed in every session. Each session concludes with a 15-min audio-guided relaxation exercise, alternating exercises that include aspects from progressive muscle relaxation, body scan, and guided imagery.

#### Specific module—low physical functioning

Participants who report low physical functioning as their most burdensome side effect perform a third Base Module session, in line with the ACSM exercise guidelines [7].

#### Specific module—CIPN

Participants who report CIPN as their most burdensome side effect receive this module as their third exercise session per week. For these participants, the exercise kit also includes a foam pad, tennis ball, golf ball, massage ball, and therapeutic clay. The content of the module is based on emerging evidence and experiences from routine care that suggest beneficial effects of sensorimotor and balance exercises on CIPN [22, 24, 25]. Each session starts with a 5–10-min warm up, including mobilization, foot muscle activation, and body awareness exercises. The core of the session consists of balance and hand training (30 min) and agility training (15 min). The balance and hand training involves 4 rounds of static balance exercises and 4 rounds of hand exercises to improve fine-motor skills, proprioception and grip strength. The agility training involves 4 rounds of agility exercises. Each session is concluded with a 5–10-min cool down, including stretching exercises and massages with a ball. Participants in this module are also encouraged to perform some exercises of the Base Module with bare feet to provide extra sensorimotor stimulation.

#### Educational component

In addition to the live-remote exercise program, the intervention includes an educational component (Fig. 2). Educational resources are delivered via the app and a printout. The overall goal of the educational component is to improve exercise adherence throughout the intervention. In addition, side effect specific educational topics are included to improve the participants’ knowledge of their side effects, enable them to understand why exercise is important, promote behavior change, and support them in maintaining a physically active lifestyle post-intervention.

### Wait list control group

Participants randomized to the wait list control group are asked to maintain their current physical activity behavior for the duration of the “control” period (12 weeks). They receive an activity tracker at baseline. After completion of the 12-week assessment, they are provided with the live-remote exercise program, following the same procedures as the intervention group. The most burdensome side effect as determined at baseline is checked with the participant again before the start of the exercise program, and a change is possible if symptoms have changed.

#### Sensor-based guidance (sub-study)

This sub-study primarily aims to evaluate the feasibility of integrating real-time wearable sensor data into live-remote exercise sessions. The objective is to complement the live-remote supervision with objective sensor data, enabling better informed exercise guidance and prescription. During the Base Module exercise session, participants wear electrophysiological sensors (Hexoskin) and an inertial measurement unit (Enode Pro) to assess movement parameters. During aerobic exercise, heart rate, step cadence, and breathing rate are monitored. During resistance exercise, repetition count and the mean concentric movement velocity of each repetition are tracked to guide resistance training based on the individual velocity loss. These data streams are available to the exercise professional in real-time, allowing them to adjust and cue exercise intensity accordingly. Additionally, nocturnal heart rate variability and sleep metrics are tracked following exercise sessions to retrospectively evaluate recovery. Participation in this sub-study is offered to wait list control group participants recruited at the German sites and is not mandatory. Participants who agree to take part in the sub-study exercise in dedicated training sessions that only involve participants wearing sensors. In case participants decline to participate in this sub-study, they proceed with the regular exercise program.

### Study outcomes

Participants visit the study center for assessments at baseline, 12 weeks, and 24 weeks (wait list control group only), with 12 weeks being the primary time point. Patient-reported outcomes are assessed using online questionnaires, which participants either receive via Castor EDC shortly prior to the study center visit or complete during their visit, prior to the physical tests. Patient-reported outcomes are also assessed remotely at 6 weeks, 18 weeks (wait list control group only), 24 weeks (intervention group only), and 36 weeks. Additionally, participants receive phone calls at these time points to collect information on potential (serious) adverse events ((S)AEs), changes in medication, and health status (e.g., cancer recurrence). During the in-person visits, anthropometrics and physical fitness/performance are assessed and blood samples are drawn. To inform whether the intervention could be delivered entirely live-remote in routine clinical practice, we assess if live-remote testing is also feasible. Therefore, we perform live-remote physical fitness and function testing in all wait list control participants at 12 and 24 weeks as well.

Socio-demographic data are assessed at baseline with a study-specific questionnaire. Medical data are based on patient-report or retrieved from medical records. Personal data are coded and all data are handled in compliance with the General Data Protection Regulation (GDPR) (EU) 2016/679. Validity of the data is checked by an independent monitor.

### Primary outcomes

The LION-RCT has two primary outcomes: HRQoL and the participant’s most burdensome side effect (i.e., cancer-related fatigue, emotional distress, low physical functioning, or CIPN).

#### HRQoL

HRQoL is assessed with the core questionnaire of the European Organization for Research and Treatment of Cancer (EORTC) that has been developed and validated for assessing HRQoL in patients with cancer (EORTC QLQ-C30). The summary score, which includes all original EORTC QLQ-C30 subscales excluding the global QoL score and financial difficulties score, is used as primary outcome [26, 27]. Scores range from 0 to 100 with a higher score indicating a better HRQoL.

#### Most burdensome side effect

The following questionnaires will be used to assess the side effects. Details on how the scores will be combined are provided in the statistical analyses section.

Cancer-related physical fatigue is assessed with the EORTC QLQ-FA12 questionnaire [28]. This is a 12-item questionnaire that assesses different dimensions of cancer-related fatigue. We use the physical fatigue dimension as primary outcome. Scores range from 0 to 100 with a higher score indicating higher levels of physical fatigue. Emotional distress is assessed with the Patient Health Questionnaire Anxiety and Depression Scale (PHQ-ADS) [19], which combines the 9-item Patient Health Questionnaire depression scale (PHQ-9) and 7-item Generalized Anxiety Disorder scale (GAD-7). A composite score, ranging from 0 to 48, is used to assess the overall burden of anxiety and depression, with a higher score indicating a higher burden.

Physical functioning is measured using the 5-item EORTC QLQ-C30 physical functioning scale. To increase reliability for use in patients with higher levels of physical functioning, five items from the EORTC questionnaire item bank were added to the physical function scale (see *Additional file 2* for the specific items). A domain-specific T-score will be calculated using the EORTC software and including all ten items. This T-score reflects the score of the participant relative to an age- and gender-matched European reference population, with 50 representing average physical functioning.

CIPN is assessed using the EORTC QLQ-CIPN20 questionnaire, which consists of 20 items addressing symptoms and the functional impact of CIPN [29]. A sum score of items 1–18, ranging from 0 to 100, is used to assess the overall CIPN burden, with a higher score indicating more severe symptoms [30].

The primary outcomes are assessed at all time points using the above-mentioned questionnaires. The same questionnaires are used to ascertain eligibility, except for CIPN (2 PRO-CTCAE items). Therefore, at baseline, they are only completed once, if the time window between eligibility screening and the baseline visit is less than 4 weeks. If the time window between eligibility screening and the baseline visit is more than 6 weeks, the participants are asked to repeat the questionnaires (Table 3).

**Table 3:** Overview of all assessments in the LION-RCT

		T0	T1	T2	T3	T4	T5
**Outcomes**	**Instrument**	Baseline	6 wks	12 wks	18 wks	24 wks	36 wks
**Primary outcomes**							
**Health-related quality of life**	EORTC QLQ-C30	X	X	X	X (WCG)	X	X
**Cancer-related fatigue**	EORTC QLQ-FA12	X	X	X	X (WCG)	X	X
**Emotional distress**	Patient Health Questionnaire Anxiety and Depression Scale (PHQ-ADS)	X	X	X	X (WCG)	X	X
**Physical function**	Modified from EORTC QLQ-C30	X	X	X	X (WCG)	X	X
**Chemotherapy-induced peripheral neuropathy**	EORTC QLQ-CIPN20	X	X	X	X (WCG)	X	X
**Secondary outcomes**							
**Patient-reported outcomes**							
**Sleep**	Pittsburgh Sleep Quality Index (PSQI)	X		X		X	X
**Pain**	Items from EORTC QLQ-SURV100	X		X		X	X
**Cognitive functioning**	2 subscales of FACT-Cog	X		X		X	X
**Body Image**	Body Image Scale	X		X		X	X
**Fear of recurrence**	Subscale of EORTC QLQ-SURV100	X		X		X	X
**Work limitations**	Work Limitations Questionnaire (WLQ)	X		X		X	X
**Self-reported physical activity**	Modified version of the GODIN questionnaire	X		X		X	X
**Quality adjusted life years**	EQ-5D-5L	X		X		X	X
**Healthcare resource consumption**	Medical Consumption Questionnaire (iMCQ)	X		X		X	X
**Productivity loss**	Productivity Cost Questionnaire (iPCQ)	X		X		X	X
**Satisfaction with the exercise program**	Self-developed questionnaire			X (IG)		X (WCG)	
**Participant insights**	Concept mapping			X (IG)		X (WCG)	
**Physical measurements (in-person)**							
**Functional performance**	Single leg stance^a^, Timed Up and Go Test	X		X		X (WCG)	
**Physical fitness**	Steep Ramp Test (SRT), Chester Step Test, 30 s sit-to-stand test, handgrip strength test, h1-RM chest- and leg press, isokinetic and isometric peak torque^b^	X		X		X (WCG)	
**Measured physical activity**	Physical activity tracker (Fitbit Inspire 3)	X	X	X	X	X	X
**Anthropometry**	Body weight, height, waist-to-hip ratio	X		X		X (WCG)	
**Resting heart rate and blood pressure**	-	X		X		X (WCG)	
**Body composition**	Bio-impedance^c^	X		X		X (WCG)	
**Blood markers**	Plasma, serum and white blood cell count	X		X			
**Physical measurements (live-remote)**							
**Functional performance**	Single leg stance, Timed Up and Go Test			X (WCG)		X (WCG)	
**Physical fitness**	Chester Step Test, 30 s sit-to-stand test, push-up test and plank position holding time test			X (WCG)		X (WCG)	
**Socio-demographical and clinical data**							
**Socio-demographics**	Self-developed questionnaire	X					
**Social support**	Index of Sojourner Social Support (ISSS)	X					
**Self-efficacy**	General Self-efficacy Scale	X					
**Cancer characteristics and treatment history**	Medical records, self-report	X					
**Medical history and concomitant diseases**	Medical records, self-report	X					
**Concomitant medication**	Medical records	X	X	X	X	X	X
**Exercise-related adverse events**	Reports of participants, exercise professionals, or medical records	X	X	X	X	X	X

*IG* intervention group, *WCG* wait list control group

^a^This test is performed on a force plate at AICSO and UKHD/DKFZ.

^b^This test is performed at UKHD/DKFZ only.

^c^This test is performed at UMCU, NKI, UKHD/DKFZ, DSHS, AICSO, ONK, CAB only.

### Secondary outcomes

#### Patient-reported outcomes

Secondary outcome measures comprise the EORTC QLQ-C30 function and symptom scales, and the other fatigue dimensions of the EORTC QLQ-FA12 (emotional, cognitive, and total fatigue scores). We extended the social and role functioning scales of the EORTC QLQ-C30 by 2 items from the EORTC item bank (see *Additional file 2* for the specific items). Sleep problems are assessed using the Pittsburgh Sleep Quality Index (PSQI), which contains 19 self-reported items assessing subjective sleep quality, sleep latency, sleep duration, habitual sleep efficiency, sleep disturbances, use of sleeping medication, and daytime dysfunction over the past month [31]. In addition to the general pain score of the EORTC QLQ-C30, pain (i.e., joint and muscle pain) is evaluated using two specific items from the EORTC Survivorship questionnaire (EORTC QLQ-SURV100; see *Additional file 2*) [32]. These items are scored on a scale from 0 to 100, with higher scores indicating more pain, and will be analyzed separately. Fear of recurrence is assessed using five specific items from the EORTC QLQ-SURV100 (see *Additional file 2*). These items are summarized into two scores, health distress and symptom awareness, with a score ranging from 0 to 100 and a higher score indicating more fear of recurrence. Cognitive functioning is assessed with two subscales of the Functional Assessment of Cancer Therapy–cognitive functioning questionnaire (FACT-cog) [33]. The “Perceived Cognitive Impairment” subscale includes 20 items with a score ranging from 0 to 80. The “Impact on QoL” subscale includes 4 items with a score ranging from 0 to 16. A higher score indicates a better QoL. The 25-item Work Limitation Questionnaire (WLQ) assesses the extent to which health problems affect specific aspects of job performance and its impact on productivity [34]. An overall WLQ-index score can be calculated. This score ranges from 0 to 100 with a higher score indicating more impact of health problems on productivity. Body image is assessed using the 10-item Body Image Scale (BIS) [35]. A total score can be calculated, ranging from 0 to 30 with a higher score indicating more body image distress.

#### Physical activity

Self-reported physical activity levels are measured using the Modified Godin-Shephard Leisure Time Exercise Questionnaire, which consists of four items that assess the average frequency and duration of mild, moderate, and vigorous intensity aerobic activities, as well as moderate-to-vigorous intensity resistance exercises performed in bouts of at least 10 min during leisure time in a typical week [36, 37]. In addition, questions were added about exercise types and settings. The activity tracker is used to measure step count, heart rate, and physical activity minutes. Participants in both the intervention and wait list control group are instructed to wear the activity tracker throughout the study period, but at least during the 7 days following randomization and the 7 days preceding the 12-week, 24-week, and 36-week post-baseline assessments. Additionally, participants are asked to wear the activity tracker during live-remote exercise sessions and physical fitness testing.

#### Anthropometrics

Anthropometric data (i.e., body weight, height, waist-to-hip ratio) are measured in lightweight clothing without shoes.

#### Body composition

As add-on measurement, body composition (fat mass and fat free mass) is assessed in some centers using a whole body/segmental bio impedance analysis (BIA). Measurements are taken in a fasted state (no enteral intake for a minimum of 2 h), with an empty bladder, in a standing or lying position. BIA devices may differ between centers, but participants within each center are consistently measured on the same device.

#### Resting heart rate and blood pressure

Resting heart rate and blood pressure are measured in a seated position prior to the physical measurements.

#### Blood markers

Blood sampling is performed by vein puncture at baseline and 12 weeks post-baseline. Plasma and serum are derived from whole blood samples. Less than 30 ml of blood is collected per visit. In the 24 h prior to blood sampling, participants are instructed not to exercise vigorously or drink alcohol, and in the 2 h prior to blood sampling, they are asked to abstain from cigarettes, food, and drinks. Immediately after collection, a proportion of the blood sample is analyzed to assess full blood count indices such as leukocytes, hemoglobin, and C-reactive protein. The remaining blood is centrifuged and stored at < −70 °C at the local laboratories according to standardized procedures. Blood samples are transferred to the central biobank at the KI for analysis after the last sample has been collected locally.

#### Physical fitness tests (in-person)

The order of the in-person physical fitness tests is standardized. First, we assess balance and functional mobility and subsequently, functional and maximal muscle strength, aerobic capacity and maximum short exercise capacity (Table 3). All tests are performed according to standardized protocols. These protocols can be found in *Additional file 3*.

#### Physical fitness tests (live-remote)

The live-remote testing sessions are performed via Zoom. Prior to conducting the physical assessments, the researcher checks whether the participant’s testing environment is suitable and safe to conduct the tests. In addition, safety measures (i.e., recording participant’s address and emergency contact details) are discussed with the participant. The live-remote testing sessions are recorded to verify the correct execution of the tests after the sessions. For the different tests, the researcher instructs the participant to change the positioning of the camera to ensure an optimal view.

At the start of the live-remote testing sessions, the participant’s heart rate is measured using the activity tracker. The order of the tests is standardized. First, we assess balance and functional mobility and subsequently, muscle strength and aerobic capacity. All tests that overlap with the in-person setting (i.e., single leg stance, Timed Up and Go test, 30 s sit-to-stand, and Chester Step Test) are conducted according to the same standard operating procedures, as described in Additional file 3*.*

Additionally, a push-up test and a plank position holding time test is performed during the live-remote testing sessions to assess muscle strength [38, 39]. The positions of these exercise are demonstrated during the in-person visits. For the push-up test, participants are asked to complete as many push-ups on knees as possible, while maintaining proper form. The test is stopped when the participant is fatigued and stops the test or fails to maintain proper form. The number of correctly performed push-ups is recorded.

For the plank position holding time test, participants are asked to hold a static plank position as long as possible. The test is stopped when the participant is fatigued and stops the test or fails to maintain proper form. The time in plank position is recorded.

#### Cost-effectiveness

A cost-effectiveness analysis (CEA) will be conducted alongside the trial by comparing the costs and effects of the intervention. The EQ-5D-5L is used to assess effects on health across five dimensions: mobility, self-care, usual activities, pain/discomfort, and anxiety/depression, each rated on five levels of severity [40]. This questionnaire is used to calculate quality-adjusted life years (QALYs). The analysis will be conducted from a societal perspective, including healthcare costs, patient and family costs, and productivity costs. Costs of the live-remote exercise program will be calculated bottom-up. Other direct costs are collected through questionnaires. A healthcare use questionnaire is used, which is based on the iMTA Medical Cost Questionnaire (iMCQ), including cost categories relevant to cancer survivors. Productivity losses are assessed using the modified Productivity Cost Questionnaire (iPCQ) [41].

#### Adherence

Adherence incorporates both attendance to the live-remote exercise sessions and compliance with the exercise prescription according to protocol. For each scheduled session, the exercise professional documents attendance in a case report form and records reasons for missed training sessions. An app (Castor Connect App) is used to capture compliance with all parts of the exercise program. Study participants record all exercise specifications (i.e., number of repetitions/rounds and, if applicable the weight or resistance band used, per exercise) and, if applicable, reasons for deviations from the protocol in the app. If participants prefer, they can also register compliance data on a paper form, in which case the data is entered into the app by the exercise trainer or study team.

#### Satisfaction

At 12 weeks (intervention group) and 24 weeks (wait list control group), we assess satisfaction with the live-remote exercise program, the exercise professionals, the activity tracker, educational resources and supporting exercise app by means of a study-specific questionnaire. In addition, participants who have completed the intervention are invited to provide more in-depth input on how the interaction between participants and the interaction between the exercise professional and participants could be optimized. This is done through a concept mapping approach. Participants can share their insights by either responding to two focus prompts, or by sorting and ranking the responses of others to these focus prompts. Responses are collected through the GroupWisdom application (Ithaca, NY, USA).

#### Sensor-based outcomes

The sensor-based sub-study will assess the feasibility, usability, and subjective experience with the technology and sensor-based exercise guidance, alongside adherence and technological reliability. In this context, usability and the experience of participants and exercise professionals regarding the technology use and sensor-based guidance are assessed using the System Usability Scale (SUS) and semi-structured interviews. Adherence is evaluated based on participant compliance with wearing the sensors (i.e., number of times sensors were worn during and after sessions). Additionally, adherence to the exercise prescription is analyzed, including the time spent within the prescribed heart rate zone (65–75%) during aerobic exercise, and the number of repetitions during resistance exercise. Technological feasibility is assessed through indicators such as device failures, connection errors, and problems documented by participants and exercise professionals.

#### Safety

All (serious) adverse events ((S)AE) are recorded according to the Exercise Harms Reporting Method (ExHaRM) [42]. First, (S)AEs are *monitored and identified* as follows: (1) Participants in both groups are asked by the study personnel about (S)AEs in a standardized manner during all follow-up visits; (2) Participants are asked by their exercise professional, before and after each live-remote exercise session, whether any (S)AEs occurred during or since the last exercise session; (3) Exercise professionals may observe a (S)AE during the exercise program; (4) (S)AE is spontaneously reported by participant between follow-up visits. Second, all (S)AEs, whether or not related to the study, are *assessed and recorded* by the exercise professional. The exercise professional indicates whether or not each event is related to the study. If unrelated, the adverse event is simply noted. If related, a detailed adverse event form is completed, including questions about the setting where the event occurred, its severity and actions taken (e.g., discontinuation or modification of intervention). Third, all (S)AEs are *reviewed* by an internal review board for relatedness to the exercise program or to the study measurements. All adverse events are coded using the Medical Dictionary for Regulatory Activities (MedDRA) to ensure standardized classification and grouping of similar events. SAEs are reported to the accredited ethical committee that approved the protocol, according to the requirements of that ethical committee.

### Sample size

The sample size is based on the expected minimal improvement of either or both primary outcomes, i.e., HRQoL or the participant’s most burdensome side effect, from baseline to 12 weeks post-baseline relative to wait list control. To adjust for multiple testing, the Bonferroni-Holm method will be used. A Cochrane meta-analysis on RCTs with exercise interventions in breast cancer survivors after adjuvant chemo- and/or radiotherapy reported an effect size (95% CI) of 0.39 (0.21–0.57) for HRQoL and 0.34 (0.05–0.62) for depression, 0.33 (0.18–0.49) for perceived physical function, and 0.32 (0.18–0.47) for fatigue [43]. The subgroup of studies with combined aerobic and resistance exercises showed a higher effect size of 0.63 (0.08–1.19) for HRQoL and 0.47 (0.02–0.92) for fatigue. A meta-analysis comprising patients with mixed cancer entities after end of treatment also showed an effect size of 0.47 (0.29–0.65) for combined exercise on fatigue [44]. Further, higher effects are typically reached in studies enrolling only patients with the targeted symptom or side effect as well as with supervised compared to unsupervised exercise interventions [9, 11]. Hence, our planned intervention is expected to have effects that are in the higher range of previously reported studies. We performed a simulation study to calculate the power of the study considering that two primary outcomes will be tested and that a meta-analytic procedure will be used to obtain an average standardized effect size for participant’s most burdensome side effects. We assumed an effect size of 0.35 to obtain a conservative estimate for the required sample size. In the simulations, we assumed a correlation of 0.3 between the two primary outcomes. In case of a common effect size of 0.35 for HRQoL and participant’s most burdensome side effects in all four strata, a sample size of 280 individuals living beyond primary curative cancer treatment (*n* = 140 per study group) was found to yield 80% power to detect an effect on each individual primary outcome, with an overall alpha of 0.05. To account for a potential drop-out of around 20%, a total number of 352 individuals will be enrolled (*n* = 176 per study group). This sample size will also facilitate exploratory moderator and subgroup analyses to better understand which individuals benefit most from exercise.

#### Statistical analysis

Descriptive statistics will be used to characterize the study population at baseline. Questionnaire scores will be calculated according to published scoring manuals. All analyses will be performed according to the intention-to-treat principle, including all randomized participants in the groups to which they were assigned, irrespective of adherence.

A positive result at the 12-week assessment of any of the two primary outcomes (i.e., HRQoL and the participant’s most burdensome side effect) is of main interest. Statistical testing for the intervention effect will be done separately for each primary outcome. HRQoL will be assessed using the EORTC QLQ-C30 summary score. For the participant’s most burdensome side effect, a meta-analytic procedure will be used to obtain an average standardized effect size (ES) (see below for further details). To adjust for multiple testing, the Bonferroni-Holm method will be used to maintain an overall alpha level of 5%. Two-sided 95% confidence intervals for the effect estimates for both primary outcomes will be presented.

A mixed model for repeated measures will be used to assess the intervention effect on the primary outcome HRQoL, while taking the hierarchical structure of the data into account (participants nested within countries). Models will be adjusted for the baseline value of the outcome and stratification factors (i.e., sex, country and the participant’s most burdensome side effect) and will include participants for whom the outcome is observed at two or more timepoints. Time since completion of curative treatment will be added as a cofactor, if there is an imbalance between groups. The primary contrast will be the comparison of the mean outcomes at 12 weeks between the intervention and control group adjusted for the baseline value.

As the participant’s most burdensome side effect will differ between participants, inference for this primary endpoint in the whole study sample will be based on a pooled estimate of the stratum-specific standardized mean differences. A two-step procedure will be used. First, standardized ES will be calculated in the four strata separately (e.g., for all patients with fatigue as main side effect, an ES will be calculated). Subsequently, these four standardized ES’s will be pooled using a fixed-effect meta-analytic model. This pooled standardized ES can be interpreted as a stratum-size weighted average of the standardized ES for participant’s most burdensome side effect at 12 weeks.

The same analysis as for HRQoL will be performed for secondary outcomes assessed at more than 2 time points. Outcomes assessed at only two time points (i.e., physical fitness and performance) will be analyzed as between-group differences in outcomes using ANCOVA, adjusted for baseline values and stratification factors. All secondary analyses will be considered exploratory with statistical testing being done at the 5% significance level. For all secondary outcomes, ESs and 95% CIs are reported without *p*-values, according to the European Medicines Agency (EMA) guidelines. The statistical analysis plan is included in the IRB study protocol.

To assess whether a similar intervention effect is seen in the whole study sample or whether subgroups can be identified that benefit most from the intervention, potential moderators of the exercise effect will be investigated, e.g., for main side effect groups, sex, cancer type, or baseline fitness level. Further, to explore potential underlying mechanisms (e.g., inflammation) of exercise effects on the primary outcomes, mediation analyses will be performed.

Missing values of the primary outcome variables as well as all other patient-reported outcomes will be assumed to be Missing at Random (MAR) for primary analyses and dealt with using mixed models for repeated measures (HQRL) and multiple imputation (most burdensome side effect). Intercurrent events (e.g., death, cancer recurrence, non-adherence (i.e., < 80% of scheduled sessions due to external reasons), contamination) will be handled according to our study protocol.

### Cost-utility analysis

In the cost-effectiveness evaluation, the net costs and effects incurred with both strategies (live-remote exercise program vs wait list control) will be compared at 12 weeks post-baseline (i.e., primary time point). The results of both cost and effect measurements will be integrated using cost-utility analyses. The analysis will be performed from a societal perspective with a time horizon of 12 weeks. Because of the short time horizon, discounting is considered redundant. In the cost-utility analysis, efficiency is expressed in terms of costs per QALY. Incremental costs and incremental effects, expressed in a ratio will be estimated. A probabilistic uncertainty analysis using bootstrapping will be performed. As it is unlikely that the full pay-back period of investments of live-remote training will be visible after a follow-up of 12 weeks, we will extrapolate findings of the economic evaluation alongside in a model-based study and determine the potential cost-effectiveness at 36 weeks and a lifetime time horizon. For this model-based study, we will combine the trial data with literature data and expert opinion scenarios.

### Analysis of live-remote testing

Agreement between live-remote and in-person tests will be determined with a Bland and Altman analysis and by using intraclass correlations (ICC) two-way mixed-effects models with absolute agreement since each measurement from the participant will be rated by the same assessor. Interpretations of the ICCs will be based on Koo and Li’s guidelines [45]: ICC values < 0.5 = poor reliability; 0.5–0.75 = moderate reliability; 0.75–0.9 = good reliability; and > 0.9 = excellent reliability.

### Analysis of the sensor-based sub-study

Feasibility outcomes related to usability and experience scores will be presented as mean ± SD along with 95% confidence intervals. Qualitative data collected from semi-structured interviews with participants and exercise professionals will be analyzed using thematic analysis to explore their subjective experiences, perceptions of usability, and detailed feedback regarding the feasibility of the technology and sensor-based exercise guidance.

### Concept mapping analysis

Statements generated and ranked by participants will be further analyzed using cluster analysis. This results in a visual map of related aspects, aggregated at a group level. This so-called “point-cluster” map is then used to identify and name overarching concepts. For each overarching concept, researchers will translate statements into actionable features and rank these according to importance and feasibility. This results in a “Go-zone map” per focus area of interest (i.e., group interaction and patient-trainer interaction), identifying which features are both feasible and important to pursue for optimizing delivery of the live-remote exercise intervention in future implementation.

## Discussion

In the current trial, we are investigating the (cost-)effectiveness of side effect-targeted, live-remote exercise for individuals living beyond primary curative cancer treatment on HRQoL and the participants’ most burdensome side effects. What makes this trial innovative is its use of live-remote exercise delivery, allowing real-time personalized support and feedback regardless of geographic location. The trial targets patients in need of (exercise) support. Our exercise program targets the participant’s most burdensome side effect, ensuring that the intervention is tailored to each participant’s specific needs, which better reflects clinical practice compared to a uniform exercise program.

We set out to address this aim through an international, multicenter RCT with a super umbrella design, i.e., combining a basket trial (i.e., participants with different side effects are all receiving the same exercise main module) with an umbrella trial (i.e., participants receive additional exercise modules targeted to their most burdensome side effect irrespective of cancer type). We chose to conduct a superiority trial rather than a non-inferiority trial with an in-person supervised exercise program as the comparator, as the latter would significantly reduce feasibility due to the large sample size required. Moreover, our aim is not to replace in-person supervised exercise programs. Instead, by comparing our live-remote exercise program to a control group, we aim to demonstrate that live-remote exercise can also be a suitable and effective option for individuals living beyond primary curative cancer treatment. Our control group is a wait list control group that receives the same live-remote exercise program after primary outcome assessment. A wait list control group was included for ethical reasons, since the current evidence suggests that all cancer survivors who experience side effects should have access to exercise support. In addition, it provides the unique opportunity to test the feasibility of remote physical fitness testing and remote sensor-based exercise guidance without compromising the validity of the RCT. We consider the additional wait time for the control group to be ethically sound, since meta-analyses have not found evidence that the timing of exercise interventions moderate their success [9–11].

This is an international study, which is conducted across geographically and culturally diverse countries in Northern, Central, and Southern Europe, and in Australia. These regions vary in terms of physical exercise promotion and support for cancer survivors. As such, this study is well-positioned to enhance external validity and support broader generalizability of the findings across different healthcare contexts.

To allow for initial natural recovery, only individuals who completed their curative treatment at least 3 months ago are eligible for study participation. Further, they need to be recruited within 1 year of completing their primary treatment, since side effects that persist longer than 1 year may have established chronically and require more complex multidisciplinary treatment.

Over the past decade, exercise-oncology trials have provided strong evidence supporting the beneficial effects of supervised exercise on HRQoL, fatigue, anxiety and depressive symptoms and low perceived physical functioning. For these outcomes, the American College of Sports Medicine (ACSM) published exercise recommendations in terms of frequency, intensity, time and type of training (FITT criteria) to individualize exercise prescription [7]. These recommendations have formed the basis of our live-remote exercise program. The four specific modules were chosen based on the most common side effects of cancer and its treatment. Apart from CIPN, sufficient evidence shows that these side effects can be addressed through exercise. CIPN was included because emerging evidence suggests that exercise, particularly sensorimotor training, may help reduce CIPN [24].

HRQoL and the participant’s most burdensome side effect are analyzed as separate co-primary outcomes. However, some conceptual overlap may exist between them. For instance, the HRQoL summary score also includes a component on fatigue, one of the potential most burdensome side effects, although with less detail than the chosen outcome measure for fatigue for the co-primary analyses. Nevertheless, by analyzing both outcomes separately and applying appropriate correction for multiple testing, we aim to assess the effect of a live-remote exercise program on both HRQoL as well as on the side effect that each participant considers most burdensome.

In exercise-oncology trials, exercise effects may be impacted by low adherence to the intervention and contamination in the control group. To improve attendance and compliance, our exercise sessions are tailored to individual needs, supervised by upskilled exercise professionals, and delivered live-remote via Zoom. This approach enables participants to join sessions from their homes or another location of their choice, minimizing barriers such as travel distance and time constraints that often hinder in-person attendance. Additionally, we closely monitor adherence and compliance in the study. Since it is impossible to blind participants in exercise-oncology trials to their respective study group, the risk of contamination is increased. To decrease this risk, we take several measures, i.e., we include relatively inactive cancer survivors and have a wait list control group. A systematic review showed that offering an exercise program after the intervention period is associated with less contamination and lower drop-out rates in the control group [46]. In addition, we clearly explain the randomization procedure to avoid disappointment when being randomized to the wait list control group and provide an activity tracker. Although providing the activity tracker may increase physical activity levels, research indicates that offering something to a control group helps to lower risk of contamination [46]. Additionally, the activity tracker will provide insight into the degree of contamination.

In summary, in the LION-RCT we examine the effects of a personalized, live-remote exercise program for individuals experiencing persistent side effects following cancer treatment and in need of supportive care. Exercise effects on HRQoL and the individual’s most burdensome side effect, as well as a range of other health outcomes will be assessed. If live-remote exercise after curative cancer treatment is proven to be (cost-)effective, implementing live-remote exercise in addition to in-person exercise would be a logical next step. This will improve the accessibility and health equity of exercise-based supportive care strategies for all cancer survivors, including those living in rural and remote areas. The results of our study can inform delivery of live-remote training and testing, which is of relevance for clinical practice and future studies. Furthermore, it can also inform international guidelines with respect to the role of live-remote exercise in improving HRQoL and reducing cancer- and treatment-related side effects in cancer survivors.

## Supplementary Information

Additional file 1

Additional file 2

Additional file 3

Additional file 4

## Data Availability

Once the PREFERABLE II project has been fully completed, the database will be anonymized and shared using DataverseNL.

## Abbreviations

ACSM: American College of Sports Medicine
BIS: Body Image Scale
CIPN: Chemotherapy-induced peripheral neuropathy
ECOG: Eastern Cooperative Oncology Group
EMA: European Medicines Agency
EORTC: European Organization for Research and Treatment of Cancer
EQ-5D-5L: EuroQol five-dimensional five-level questionnaire
ExHaRM: Exercise Harms Reporting Method
FACT-cog: Functional Assessment of Cancer Therapy Cognitive Function
HRQoL: Health-Related Quality of Life
iMCQ: iMTA Medical Cost Questionnaire
iPCQ: iMTA Productivity Cost Questionnaire
PHQ-ADS: Patient Health Questionnaire Anxiety and Depression Scale
PRO-CTCAE: Patient-Reported Outcomes version of the Common Terminology Criteria for Adverse Events
PSQI: Pittsburgh Sleep Quality Index
QoL: Quality of Life
RCT: Randomized Controlled Trial
SRT: Steep Ramp Test
WLQ: Work Limitations Questionnaire

## References

[1] Burgess J, Ferdousi M, Gosal D, et al. Chemotherapy-induced peripheral neuropathy: epidemiology, pathomechanisms and treatment. Oncol Ther. 2021;9(2):385–450. 10.1007/s40487-021-00168-y. 34655433 10.1007/s40487-021-00168-y PMC8593126

[2] Mitchell AJ, Ferguson DW, Gill J, Paul J, Symonds P. Depression and anxiety in long-term cancer survivors compared with spouses and healthy controls: a systematic review and meta-analysis. Lancet Oncol. 2013;14(8):721–32. 10.1016/S1470-2045(13)70244-4. 23759376 10.1016/S1470-2045(13)70244-4

[3] Kang YE, Yoon JH, hyun N, Ahn YC, Lee EJ, Son CG. Prevalence of cancer-related fatigue based on severity: a systematic review and meta-analysis. Sci Rep. 2023;13(1):12815. 10.1038/s41598-023-39046-0. 37550326 10.1038/s41598-023-39046-0 PMC10406927

[4] Firkins J, Hansen L, Driessnack M, Dieckmann N. Quality of life in “chronic” cancer survivors: a meta-analysis. J Cancer Surviv. 2020;14(4):504–17. 10.1007/s11764-020-00869-9. 32162194 10.1007/s11764-020-00869-9

[5] Mustian KM, Alfano CM, Heckler C, et al. Comparison of pharmaceutical, psychological, and exercise treatments for cancer-related fatigue a meta-analysis. JAMA Oncol. 2017;3(7):961-968. https://doi.org/10.1016/S2214-109X(16)30265-0.Cost-effectiveness 10.1001/jamaoncol.2016.6914 PMC5557289 28253393

[6] Aune D, Markozannes G, Abar L, et al. Physical activity and health-related quality of life in women with breast cancer: a meta-analysis. JNCI Cancer Spectr. 2022;6(6):pkac072. 10.1093/jncics/pkac072. 36474321 10.1093/jncics/pkac072 PMC9727071

[7] Campbell KL, Winters-Stone KM, Wiskemann J, et al. Exercise guidelines for cancer survivors: consensus statement from international multidisciplinary roundtable. Med Sci Sports Exerc. 2019;51(11):2375-2390. 10.1249/MSS.0000000000002116 PMC8576825 31626055

[8] Ligibel JA, Bohlke K, May AM, et al. Exercise, diet, and weight management during cancer treatment: ASCO guideline. J Clin Oncol. 2022;40(22):2491–507. 10.1200/JCO.22.00687. 35576506 10.1200/JCO.22.00687

[9] Van Vulpen J, Sweegers M, Peeters P, et al. Moderators of exercise effects on cancer-related fatigue: a meta-analysis of individual patient data. Med Sci Sports Exerc. 2020;52(2):303–14. 10.1249/MSS.0000000000002154. 31524827 10.1249/MSS.0000000000002154 PMC6962544

[10] Sweegers MG, Altenburg TM, Brug J, et al. Effects and moderators of exercise on muscle strength, muscle function and aerobic fitness in patients with cancer: a meta-analysis of individual patient data. Br J Sports Med. 2019;53(13):812. 10.1136/bjsports-2018-09919. 30181323 10.1136/bjsports-2018-099191

[11] Buffart LM, Kalter J, Sweegers MG, et al. Effects and moderators of exercise on quality of life and physical function in patients with cancer: an individual patient data meta-analysis of 34 RCTs. Cancer Treat Rev. 2017;52:91–104. 10.1016/j.ctrv.2016.11.010. 28006694 10.1016/j.ctrv.2016.11.010

[12] Kennedy MA, Bayes S, Newton RU, et al. Implementation barriers to integrating exercise as medicine in oncology: an ecological scoping review. Journal of Cancer Survivorship. 2022;16(4):865–81. 10.1007/s11764-021-01080-0. 34510366 10.1007/s11764-021-01080-0 PMC9300485

[13] Clifford BK, Mizrahi D, Sandler CX, et al. Barriers and facilitators of exercise experienced by cancer survivors: a mixed methods systematic review. Support Care Cancer. 2018;26(3):685–700. 10.1007/s00520-017-3964-5. 29185105 10.1007/s00520-017-3964-5

[14] Gonzalo-Encabo P, Wilson RL, Kang DW, Normann AJ, Dieli-Conwright CM. Exercise oncology during and beyond the COVID-19 pandemic: are virtually supervised exercise interventions a sustainable alternative? Crit Rev Oncol Hematol. 2022;174: 103699. 10.1016/j.critrevonc.2022.103699. 35526668 10.1016/j.critrevonc.2022.103699 PMC9069989

[15] Smith GVH, Myers SA, Fujita RA, Yu C, Campbell KL. Virtually supervised exercise programs for people with cancer: a scoping review. Cancer Nurs. Published online 2024. https://journals.lww.com/cancernursingonline/fulltext/9900/virtually_supervised_exercise_programs_for_people.236.aspx 10.1097/NCC.0000000000001353 38598778

[16] Crisafio ME, Anderson HAL, Thraen-Borowski KM, Schmitz KH, Leach HJ. Scoping review of videoconference online exercise programs for cancer survivors in community settings. Transl J Am Coll Sports Med. 2024;9(2). https://journals.lww.com/acsm-tj/fulltext/2024/04120/scoping_review_of_videoconference_online_exercise.6.aspx

[17] Riebe D, Franklin BA, Thompson PD, et al. Updating ACSM’s recommendations for exercise preparticipation health screening. Med Sci Sports Exerc. 2015;47(11):2473–9. 10.1249/MSS.0000000000000664. 26473759 10.1249/MSS.0000000000000664

[18] Giesinger JM, Loth FLC, Aaronson NK, et al. Thresholds for clinical importance were established to improve interpretation of the EORTC QLQ-C30 in clinical practice and research. J Clin Epidemiol. 2020;118:1-8. 10.1016/j.jclinepi.2019.10.003 10.1016/j.jclinepi.2019.10.003 31639445

[19] Kroenke K, Wu J, Yu Z, et al. Patient health questionnaire anxiety and depression scale: initial validation in three clinical trials. Psychosom Med. 2016;78(6):716-727. 10.1097/PSY.0000000000000322 10.1097/PSY.0000000000000322 PMC4927366 27187854

[20] Knoerl R, Mazzola E, Mitchell SA, et al. Measurement properties of brief neuropathy screening items in cancer patients receiving taxanes, platinums, or proteasome inhibitors. J Patient Rep Outcomes. 2021;5(1):101. 10.1186/s41687-021-00377-z. 34568984 10.1186/s41687-021-00377-z PMC8473487

[21] Hiensch AE, Monninkhof EM, Schmidt ME, et al. Design of a multinational randomized controlled trial to assess the effects of structured and individualized exercise in patients with metastatic breast cancer on fatigue and quality of life: the EFFECT study. Trials. 2022;23(1):610. 10.1186/s13063-022-06556-7. 35906659 10.1186/s13063-022-06556-7 PMC9335464

[22] Zhou S, Chen G, Xu X, et al. Comparative efficacy of various exercise types on cancer-related fatigue for cancer survivors: a systematic review and network meta-analysis of randomized controlled trials. Cancer Med. 2025;14(7): e70816. 10.1002/cam4.70816. 40145635 10.1002/cam4.70816 PMC11948276

[23] Gonzalez M, Pascoe MC, Yang G, et al. Yoga for depression and anxiety symptoms in people with cancer: a systematic review and meta-analysis. Psychooncology. 2021;30(8):1196–208. 10.1002/pon.5671. 33763925 10.1002/pon.5671

[24] Streckmann F, Elter T, Lehmann HC, et al. Preventive effect of neuromuscular training on chemotherapy-induced neuropathy: a randomized clinical trial. JAMA Intern Med. 2024;184(9):1046–53. 10.1001/jamainternmed.2024.2354. 38949824 10.1001/jamainternmed.2024.2354 PMC11217888

[25] Kleckner IR, Park SB, Streckmann F, Wiskemann J, Hardy S, Mohile N. Systematic review of exercise for prevention and management of chemotherapy-induced peripheral neuropathy. In: Lustberg M, Loprinzi C, eds. Diagnosis, management and emerging strategies for chemotherapy-induced neuropathy: a MASCC book. Springer International Publishing; 2021:183-241. 10.1007/978-3-030-78663-2_8

[26] Giesinger JM, Kieffer JM, Fayers PM, et al. Replication and validation of higher order models demonstrated that a summary score for the EORTC QLQ-C30 is robust. J Clin Epidemiol. 2016;69:79-88. 10.1016/j.jclinepi.2015.08.007 10.1016/j.jclinepi.2015.08.007 26327487

[27] Aaronson N, Ahmedzai S, Bergman B, et al. The European Organization for Research and Treatment of Cancer QLQ-C30: a quality-of-life instrument for use in international clinical trials in oncology. J Natl Cancer Inst. 1993;85:365–76. 8433390 10.1093/jnci/85.5.365

[28] Weis J, Tomaszewski KA, Hammerlid E, et al. International psychometric validation of an EORTC quality of life module measuring cancer related fatigue (EORTC QLQ-FA12). J Natl Cancer Inst. 2017;109(5). 10.1093/jnci/djw273 10.1093/jnci/djw273 28376231

[29] Postma TJ, Aaronson NK, Heimans JJ, et al. The development of an EORTC quality of life questionnaire to assess chemotherapy-induced peripheral neuropathy: the QLQ-CIPN20. Eur J Cancer. 2005;41(8):1135–9. 10.1016/j.ejca.2005.02.012. 15911236 10.1016/j.ejca.2005.02.012

[30] Kieffer JM, Postma TJ, van de Poll-Franse L, et al. Evaluation of the psychometric properties of the EORTC chemotherapy-induced peripheral neuropathy questionnaire (QLQ-CIPN20). Quality of Life Research. 2017;26(11):2999–3010. 10.1007/s11136-017-1626-1. 28634676 10.1007/s11136-017-1626-1

[31] Buysse D, Reynolds C 3rd, Monk T, Berman S, Kupfer D. The Pittsburgh sleep quality index: a new instrument for psychiatric practice in research. Psychiatry Res. 1989;28(2):193–213. 10.1152/ajpheart.00471.2010. 2748771 10.1016/0165-1781(89)90047-4

[32] van Leeuwen M, Kieffer JM, Young TE, et al. Correction to: Phase III study of the European Organisation for Research and Treatment of Cancer Quality of Life cancer survivorship core questionnaire. J Cancer Survivorship. Published online 2022. 10.1007/s11764-022-01199-8 10.1007/s11764-022-01199-8 35277836

[33] Joly F, Lange M, Rigal O, et al. French version of the Functional Assessment of Cancer Therapy–Cognitive Function (FACT-Cog) version 3. Support Care Cancer. 2012;20(12):3297–305. 10.1007/s00520-012-1439-2. 22549504 10.1007/s00520-012-1439-2

[34] Lerner D, Amick BCIII, Rogers WH, Malspeis S, Bungay K, Cynn D. The work limitations questionnaire. Med Care. 2001;39(1). https://journals.lww.com/lww-medicalcare/Fulltext/2001/01000/The_Work_Limitations_Questionnaire.9.aspx 10.1097/00005650-200101000-00009 11176545

[35] Hopwood P, Fletcher I, Lee A, Al Ghazal S. A body image scale for use with cancer patients. Eur J Cancer. 2001;37(2):189–97. 10.1016/S0959-8049(00)00353-1. 11166145 10.1016/s0959-8049(00)00353-1

[36] Amireault S, Godin G. The Godin-Shephard leisure-time physical activity questionnaire: validity evidence supporting its use for classifying healthy adults into active and insufficiently active categories. Percept Mot Skills. 2015;120(2):604-622. 10.2466/03.27.PMS.120v19x7 10.2466/03.27.PMS.120v19x7 25799030

[37] Amireault S, Godin G, Lacombe J, Sabiston CM. Validation of the Godin-Shephard Leisure-Time Physical Activity Questionnaire classification coding system using accelerometer assessment among breast cancer survivors. J Cancer Surviv. 2015;9(3):532–40. 10.1007/s11764-015-0430-6. 25666749 10.1007/s11764-015-0430-6

[38] Crosby BJ, Newton RU, Galvão DA, et al. Feasibility of supervised telehealth exercise for patients with advanced melanoma receiving checkpoint inhibitor therapy. Cancer Med. 2023;n/a(n/a). 10.1002/cam4.6091 10.1002/cam4.6091 PMC10358269 37184115

[39] Guidarelli C, Lipps C, Stoyles S, Dieckmann NF, Winters-Stone KM. Remote administration of physical performance tests among persons with and without a cancer history: establishing reliability and agreement with in-person assessment. J Geriatr Oncol. 2022;13(5):691–7. 10.1016/j.jgo.2022.02.002. 35177378 10.1016/j.jgo.2022.02.002 PMC9232927

[40] Herdman M, Gudex C, Lloyd A, et al. Development and preliminary testing of the new five-level version of EQ-5D (EQ-5D-5L). Qual Life Res. 2011;20(10):1727-1736. 10.1007/s11136-011-9903-x 10.1007/s11136-011-9903-x PMC3220807 21479777

[41] Bouwmans C, Krol M, Severens H, Koopmanschap M, Brouwer W, Van Roijen LH. The iMTA Productivity Cost Questionnaire: a standardized instrument for measuring and valuing health-related productivity losses. Value in Health. 2015;18(6):753–8. 10.1016/j.jval.2015.05.009. 26409601 10.1016/j.jval.2015.05.009

[42] Spence RR, Sandler CX, Jones TL, McDonald N, Dunn RM, Hayes SC. Practical suggestions for harms reporting in exercise oncology: the Exercise Harms Reporting Method (ExHaRM). BMJ Open. 2022;12(12): e067998. 10.1136/bmjopen-2022-067998. 36600391 10.1136/bmjopen-2022-067998 PMC9743394

[43] Lahart IM, Metsios GS, Nevill AM, Carmichael AR. Physical activity for women with breast cancer after adjuvant therapy. Cochrane Database Syst Rev. 2018;1(1):CD011292. 10.1002/14651858.CD011292.pub2 10.1002/14651858.CD011292.pub2 PMC6491330 29376559

[44] Hilfiker R, Meichtry A, Eicher M, et al. Exercise and other non-pharmaceutical interventions for cancer-related fatigue in patients during or after cancer treatment: a systematic review incorporating an indirect-comparisons meta-analysis. Br J Sports Med. 2018;52(10):651. 10.1136/bjsports-2016-096422. 28501804 10.1136/bjsports-2016-096422 PMC5931245

[45] Koo TK, Li MY. A guideline of selecting and reporting intraclass correlation coefficients for reliability research. J Chiropr Med. 2016;15(2):155–63. 10.1016/j.jcm.2016.02.012. 27330520 10.1016/j.jcm.2016.02.012 PMC4913118

[46] Steins Bisschop CN, Courneya KS, Velthuis MJ, et al. Control group design, contamination and drop-out in exercise oncology trials: a systematic review. PLoS One. 2015;10(3): e0120996. 10.1371/journal.pone.0120996. 25815479 10.1371/journal.pone.0120996 PMC4376879

