# Supplementary material for: Effects of personalized live-remote exercise for individuals living beyond primary curative cancer treatment: study protocol for a multinational, super umbrella randomized controlled trial (LION-RCT)
Source: Trials. 2025 Nov 24;26:540. doi: 10.1186/s13063-025-09263-1 (PMC12642165; doi:10.1186/s13063-025-09263-1)
Supplement: Supplementary file 1 — Additional file 1 [file 13063_2025_9263_MOESM1_ESM.docx]

# Appendix I

Here, we provide more details with respect to organization of the PREFERABLE-LION study.

Sponsor of the PREFERABLE-LION study

University Medical Center Utrecht

Julius Center for Health Sciences and Primary Care

Heidelberglaan 100, 3584 CX Utrecht, The Netherlands
PI Prof. Dr. Anne May, Professor of Clinical Epidemiology of Cancer Survivorship

Project management

The day-to-day operational project management, coordination and oversight of the PREFERABLE-LION study is the responsibility of Prof.Dr. May, supported by researchers from the UMC Utrecht.

Study management

A Clinical Research Organization (Julius Clinical// https://www.juliusclinical.com/) is responsible for the study management of the trial. This includes the following activities: obtaining regulatory and ethical approval, site contracting, training of monitors, data management, processing and reporting of SAE’s, and monitoring at sites.

Steering committee (consisting of all Work Package (WP) leaders)

Decisions which concern changes in strategy are made by the steering committee of the PREFERABLE-LION consortium. The committee consists of the LION WP leaders and/or at least one representative of the participating centers. The steering committee is chaired by Prof. Dr. May and established under mutual agreement with the entire consortium. Decisions of the committee are made first and foremost on consensus making.

Data management

Study data is captured using an electronic data capture system (Castor EDC®). Castor® is compliant with all relevant regulations, such as ICH E6 Good Clinical Practice and the General Data Protection Regulation (GDPR). Required data for this study is obtained from the participant’s medical records/source documents or by direct entry, where the information was first recorded and then entered into the eCRF. Data from the eCRF is encoded and stored in a study database. Only authorized site staff is allowed to enter data into the eCRF and make changes to eCRF data. Additionally, data is obtained from online questionnaires completed by the participants.

The eCRFs are reviewed by a monitor from the CRO for completeness and accuracy as described in the Monitoring Plan of the study.

Data management details are described in a data management plan.

Data Safety Monitoring Board (DSMB) and interim analyses

Not applicable. We consider a DSMB not necessary since this is a low-risk trial. The safety of physical exercise has been established in previous studies in cancer survivors.

No interim analyses are planned.

Protocol amendments

Any modifications made to the protocol after receipt of the Regulatory Authorities / Ethics Committees approval will be re-submitted in accordance with local procedures and regulatory requirements. Modifications or new information that might change patients’ willingness to participate, will be communicated to the included participants.

Dissemination policy

The investigators will inform all interested participants about the main results of the study. The results of the study will be reported in peer reviewed international journals, national non-peer reviewed journals, e.g., from patient organisations and professional associations, and presented on (inter-)national conferences. Also, policy regulators, healthcare providers and other important stakeholders will be informed on the outcomes of the PREFERABLE-LION study.

Authorship eligibility guidelines

Authorship eligibility for all trial-associated publications will follow the International Committee of Medical Journal Editors (ICJME) criteria for authorship. We do not intend to use professional writers for any publications related to the PREFERABLE II-LION study.
