# Supplementary material for: Effects of personalized live-remote exercise for individuals living beyond primary curative cancer treatment: study protocol for a multinational, super umbrella randomized controlled trial (LION-RCT)
Source: Trials. 2025 Nov 24;26:540. doi: 10.1186/s13063-025-09263-1 (PMC12642165; doi:10.1186/s13063-025-09263-1)
Supplement: Supplementary file 3 — Additional file 3 [file 13063_2025_9263_MOESM3_ESM.docx]

# Additional file 2

*Additional items on physical functioning from EORTC item bank:*

| No. |  | Not at  All | A  Little | Quite  a Bit | Very  Much |
| --- | --- | --- | --- | --- | --- |
| 18 | Do you have any trouble running fast? |  |  |  |  |
| 20 | Do you have any trouble carrying heavy bags upstairs? |  |  |  |  |
| 31 | Do you have any trouble running a short distance, such as to catch the bus? |  |  |  |  |
| 8 | Do you have any trouble lifting a box weighing about  10 kg? |  |  |  |  |
| 23 | Do you have any trouble taking a long walk carrying a  heavy backpack on your back (e.g., filled rucksack)? |  |  |  |  |

*Additional items on role functioning and social functioning from EORTC item bank:*

| No. |  | Not  at All | A  Little | Quite  a Bit | Very  Much |
| --- | --- | --- | --- | --- | --- |
| 3  (RF) | Have you been limited in doing physically demanding recreational activities (e.g., swimming or cycling)? |  |  |  |  |
| 9  (RF) | Have you been limited in doing heavy housework (e.g., washing floors or vacuuming)? |  |  |  |  |
| 4  (SF) | As a result of your physical condition or medical treatment have you been less able to see your family or friends? |  |  |  |  |
| 7  (SF) | As a result of your physical condition or medical treatment, have you spent less time with your family or friends? |  |  |  |  |

*Additional items on pain selected from EORTC QLQ-SURV100:*

| No. | During the past week: | Not at  All | A  Little | Quite  a Bit | Very  Much |
| --- | --- | --- | --- | --- | --- |
| 26 | Have you had aches or pain in your joints? |  |  |  |  |
| 27 | Have you had aches or pain in your muscles? |  |  |  |  |

*Additional items on fear of recurrence selected from EORTC QLQ-SURV100:*

| No. | Because of your experience with cancer: | Not  at All | A  Little | Quite  a Bit | Very  Much |
| --- | --- | --- | --- | --- | --- |
| 65 | Are you alert for symptoms that may signal a return of  your cancer? |  |  |  |  |
| 66 | Are you more likely to contact your doctor when you  experience symptoms? |  |  |  |  |
| No. | During the past week: | Not  at All | A  Little | Quite  a Bit | Very  Much |
| 53 | Have you worried about your cancer coming back or that  it may spread to other parts of your body? |  |  |  |  |
| 54 | Have you worried about getting another type of cancer? |  |  |  |  |
| 55 | Have you worried about your health? |  |  |  |  |
