# Supplementary material for: Effects of personalized live-remote exercise for individuals living beyond primary curative cancer treatment: study protocol for a multinational, super umbrella randomized controlled trial (LION-RCT)
Source: Trials. 2025 Nov 24;26:540. doi: 10.1186/s13063-025-09263-1 (PMC12642165; doi:10.1186/s13063-025-09263-1)
Supplement: Supplementary file 4 — Additional file 4 [file 13063_2025_9263_MOESM4_ESM.docx]

# Additional file 3

## Physical fitness tests (in-person)

## Balance and Functional Mobility

Balance is measured with a single leg stance test and functional performance with the Timed up and Go test.

For the single leg stance, participants are asked to stand on one leg as long as possible for a maximum of 45 seconds in two conditions (i.e., eyes open and closed). Each condition is performed up to 3 times allowing for a 30 sec break between each attempt. The test with eyes open and closed is alternated. The test stops when the participant loses balance: arms off chest, lifted foot touches ground/standing leg, planted foot moves. If the participant reaches 45 seconds in an attempt, there is no need to perform further attempts (in that condition). The best attempt of each condition is recorded in seconds. As add-on measurement, this test is performed on a force plate in some centers (*Table 3 of main text*). With the force plate, the center of pressure can be calculated, providing information about postural stability.

The Timed up and Go test assesses functional mobility.^1^ The participant is instructed to stand up from a chair, walk 3 meters, turn and sit down again, without running. The time needed to complete the test is recorded. This test provides information about risk of falling.

Muscle strength

Upper body muscle strength is measured with a handgrip strength test and a hypothetical 1-repetition maximum (h1-RM) chest press or isokinetic dynamometer (IsoMed 2000®).

Handgrip strength is assessed using a handgrip dynamometer (hydraulic Jamar®) with participants seated, their elbow by their side and flexed at a 70° angle, and a neutral wrist position. The participant is asked to squeeze the dynamometer as hard as possible with each hand. Testing alternates between hands, and the best result from three attempts per hand is recorded.

The hypothetical one repetition maximum (h1-RM) chest press is conducted using a chest press machine. After a warm-up of 10-12 repetitions with a light weight, the assessor selects a weight that the participant is likely able to lift a maximum of 5-12 times. If the participant can lift the weight more than 12 times, the test is repeated with a heavier weight after 2 minutes of rest. The aim is to determine a RM between 5-12 within 3 attempts. The weight and number of repetitions is recoded to estimate the h1-RM using a regression equation.^2^ If no chest press machine is available, maximal isokinetic peak torque during elbow flexion and extension is tested and recorded for each arm at 60°/s with the isokinetic dynamometer. Measures of upper body strength may differ between centers, but not within centers.

Lower body muscle strength is measured with a h1-RM leg press or isokinetic dynamometer (IsoMed 2000®) and a 30 sec sit-to-stand test. The h1-RM leg press is conducted using the same protocol as the h1-RM chest press. If no leg press is available, maximal isokinetic peak torque during knee extension and flexion is tested and recorded for each leg at 60°/s with the isokinetic dynamometer. Measures of leg strength may differ between centers, but not within centers. Functional lower body muscle strength is determined with the 30 sec sit-to-stand test. The participant is asked to perform the sit-to-stand movement from a chair, with arms crossed over the chest, as many times as possible within 30 seconds.^3^ The number of completed stands is recorded.

Aerobic capacity and maximal short exercise capacity

Aerobic capacity is measured using the Chester Step Test. For the Chester Step Test, participants are instructed to step up and down an aerobic step which has a standardized height of 15 cm in a metronome-given rhythm for a maximum of 10 minutes.^4^ The test starts with a step rate of 60 steps per minute and is increased by 20 steps per minute every 2 minutes with a maximum of 5 stages (i.e., 140 steps per minute). At the end of each stage, the participants’ heart rate is recorded. The test ends when the participant reaches a heart rate of 80% of the estimated maximum heart rate (HR_max_ = 220-age), reports pain or discomfort, is unable to keep the metronome pace or successfully completes all five stages. For participants on beta-blockers, a Rate of Perceived Exertion (RPE) of 14/20 is chosen as the termination criterion. At the end of the test, the heart rate, highest level completed, total stepping time and RPE are recorded.

Maximal Short Exercise Capacity (MSEC) is measured with the Steep Ramp Test using a cycle ergometer.^5^ After 3 min of unloaded cycling, the test starts at 25 Watts and is increased by 2.5 Watts per second or 25 Watts per 10 s until exhaustion. Participants are instructed to cycle between 70 and 90 revolutions per minute (RPM). The test ends when the cycling cadence drops below 60 RPM or when the participant experiences any pain, anxiety, dizziness or nausea. After termination, the participant is asked to continue cycling at an easy cadence and with minimal load to recover. The outcome is registered as the highest achieved output in Watts and is referred to as the MSEC. From the MSEC, peak Wattage (W_peak_) can be estimated using a regression equation.^6^ Additionally, we record the RPE, time cycled, and heart rate at the end of the test as well as 1 and 2 min after termination.

References

1. Podsiadlo D, Richardson S. The Timed “Up & Go”: A Test of Basic Functional Mobility for Frail Elderly Persons. *J Am Geriatr Soc*. 1991;39(2):142-148. doi:https://doi.org/10.1111/j.1532-5415.1991.tb01616.x

2. Schneider J, Schluter K, Rosenberger F, Wiskemann J. Are percentages of the one-repetition maximum (1-RM) suitable for prescribing resistance exercise in cancer survivors? - Comparability and prediction accuracy of frequently used 1-RM testing procedures. *Preprint*. Published online October 21, 2022.

3. Jones CJ, Rikli RE, Beam WC. A 30-s Chair-Stand Test as a Measure of Lower Body Strength in Community-Residing Older Adults. *Res Q Exerc Sport*. 1999;70(2):113-119. doi:10.1080/02701367.1999.10608028

4. Bennett H, Parfitt G, Davison K, Eston R. Validity of Submaximal Step Tests to Estimate Maximal Oxygen Uptake in Healthy Adults. *Sports Medicine*. 2016;46(5):737-750. doi:10.1007/s40279-015-0445-1

5. De Backer IC, Schep G, Hoogeveen A, Vreugdenhil G, Kester AD, van Breda E. Exercise Testing and Training in a Cancer Rehabilitation Program: The Advantage of the Steep Ramp Test. *Arch Phys Med Rehabil*. 2007;88(5):610-616. doi:https://doi.org/10.1016/j.apmr.2007.02.013

6. Stuiver MM, Kampshoff CS, Persoon S, et al. Validation and Refinement of Prediction Models to Estimate Exercise Capacity in Cancer Survivors Using the Steep Ramp Test. *Arch Phys Med Rehabil*. 2017;98(11):2167-2173. doi:https://doi.org/10.1016/j.apmr.2017.02.013
